# Factors associated with poor self-rated health among chronic kidney disease patients and their health care utilization: Insights from LASI wave-1, 2017-18

**DOI:** 10.3389/fneph.2022.968285

**Published:** 2023-01-06

**Authors:** Swetalina Nayak, Soumya Ranjan Nayak, Alice Alice, Debadutta Sahoo, Srikanta Kanungo, Tanveer Rehman, Sanghamitra Pati, Subrat Kumar Palo

**Affiliations:** Department of Health Research (ICMR)-Regional Medical Research Center, Bhubaneswar, India

**Keywords:** health care utilization, self-rated health, chronic kidney disease, chronic conditions, quality of life, lasi, India

## Abstract

**Background:**

Chronic kidney disease (CKD), associated with other chronic conditions affects the physical, behavioral, and psychological aspects of an individual, leading to poor self-rated health. Hence, we aimed to assess the factors associated with poor self-rated health (SRH) in CKD patients. Additionally, we assessed their health care utilization.

**Methods:**

This is an observational study consisting of 527 CKD patients from Longitudinal Aging Study in India (LASI), 2017-2018. A descriptive statistic computed prevalence. Regression analysis assessed the association between poor SRH and socio-demographic variables presented as adjusted odds ratio with a confidence interval of 95%. Health care utilization among CKD patients was graphically presented.

**Results:**

Around 64% of CKD patients had poor SRH. Aged 75 years and above (AOR=1.8, 95% CI= 0.5-6.8), rural residents (AOR= AOR 1.8, 95% CI =1.0 -3.1) and those with other chronic conditions (AOR=5.1, 95% CI= 2.3-11.0) were associated with poor SRH. Overall 79% of the CKD patients availed health care facility, most (44.8%) of those visit private facility.

**Conclusion:**

We observed older adults, females, rural residents, and having other chronic conditions were associated with poor SRH among CKD patients which highlights the need for equitable and strengthened health care system. There is an urgent need to provide accessible, affordable and quality healthcare services for these individuals so as to maintain continuity of care.

## 1 Introduction

Chronic kidney disease (CKD) is one of the most important causes of mortality and morbidity. It was responsible for 4.6% of all-cause mortality in 2017 (1). Serious public health concerns have been raised in recent years due to the progressive rise in the prevalence and disability-adjusted life years (DALYs) associated with CKD (2, 3). Between 1990 and 2017, there was an increase in CKD prevalence and DALYs by 29.3% and 93.2%, respectively. Furthermore, it is a cause of 35.8% of all fatalities (3).

CKD has a complex and multidimensional aetiology. Chronic illnesses, including diabetes, hypertension, and obesity (4), can also lead to CKD. CKD, accompanied by other chronic conditions, often leads to a higher mortality rate, which may exacerbate patients’ out-of-pocket expenditure (OOPE), resulting in compromised health status (5, 6).

CKD results in considerable restrain and restrictions in the patient’s activities of daily living (ADL) and habituated dietary patterns. Furthermore, it impacts the health-related quality of life (HRQoL) (7, 8). HRQoL is an empirical way to assess quality of life (QoL), often used to measure self-perceived health (9). Self-rated health (SRH) is a widely used proxy indicator of HRQoL. The SRH is a measurement that study participants use to reflect how they feel about their present health. Poor SRH is an indicator of increased risk of death among renal disorder patients (10). The World Health Organization (WHO) endorses SRH as a crucial marker of population health and healthy life expectancy (11). SRH is a summarized measurement of individual-level components that can vary greatly and is the outcome of a complicated process of cognitive-emotional well-being of individuals established by the community and culture they live and follow (12). Although SRH has recently supplanted clinical evaluation criteria in surveys, our understanding of how people form their opinions of SRH and what characteristics are linked to poor SRH ratings, especially in the CKD population, is still limited.

SRH is also related to continuity of care and hence health care utilization (HCU), plays a pivotal role in meeting patients’ needs. HCU generally represents the usage of the existing health care system. It typically mirrors the survival rates of patients with chronic illnesses. Although for continuity of care, HCU is necessary, it is affected by many factors, including lower active health worker density and skewed distribution among public-private settings (13). Higher OOPE, catastrophic health expenditure (CHE), and insurance status (14) also play an important role.

Keeping an eye on the causes, consequences, and costs related to CKD, the government of India (GOI) implemented the “Pradhan Mantri National Dialysis Program (PMNDP)” for below poverty line (BPL) patients in public-private-partnership (PPP) mode (15). Additionally, sustainable development goals (SDG) have some indirect implications that could indirectly be linked with CKD (16, 17). Additionally, in India, the National Program for Prevention and Control of Cancer, Diabetes, CVD, and Stroke (NPCDCS) is also working towards the prevalence and control of the main cause of CKD. Still, the Global Burden of Disease suggests that China and India were home to roughly one-third of all CKD patients in 2017 (3), while; India alone had 115.1 million cases (3).

Given all the implications, assessing the prevalence of poor self-rated health among CKD patients and the factors associated with poor SRH is vital. However, little evidence suggests the factors associated with poor SRH. Evidence-based on the outcome would help the policy makers formulate better policies for CKD patients, focusing on specific factors. Hence, we conducted this secondary data analysis to assess the prevalence and determinants of poor SRH in CKD patients. Additionally, we aimed to assess the health care utilization among CKD patients.

## 2 Methods

### 2.1 Data source

We conducted a secondary data analysis of a nationwide baseline survey of “Longitudinal Ageing Study in India” (LASI), wave 1 conducted between 2017 -2018 (18–20). It was jointly carried out by the Harvard TH Chan School of Public Health, the International Institute for Population Sciences (IIPS), and the University of Southern California, adopting a multistage stratified area probability cluster sampling strategy (19). “LASI Eligible Households (LEH)” were the unit of observations for this survey, comprising of one or more persons dwelling in a household with at least one member aged 45 years and above (and their spouses irrespective of age). This study included 29 Indian states except for Sikkim and 6 union territory (UT), using a three-stage sample approach for rural regions while a four-stage sampling approach for urban areas (21). Furthermore, the selected houses were distributed with an individual survey questionnaire to each willing respondent aged 45 and above and their spouse (regardless of age) (18, 21, 22). The details of methods used during LASI wave-1 has been published on IIPS, Mumbai’s website (20, 21).

### 2.2 Study participants and sample size

The survey comprised of a total of 72,250 individuals from 42,949 households. Participants were asked “have you ever been diagnosed with any of the following urogenital conditions or diseases?” (options: a. Chronic renal failure, b. Incontinence, c. Kidney stones, d. BPH, e. None). Respondents who agreed to the presence of the “chronic renal failure” were recruited as the participants for this study. A total of 527 adult individuals were eligible for the current study.

## 3 Variable description

### 3.1 Outcome variable

Self-rated health among patients with CKD:

Self-rated health (SRH) was assessed by asking respondents to rate their current overall health on a five-point ordinal scale ranging; from poor, fair, good, very good, and excellent. This outcome variable was dichotomized into the poor (by combining poor and fair) and the good (by combining good, very good, and excellent) (23, 24). A binary variable was created to facilitate more meaningful interpretation, encoding 1 for “poor SRH for CKD disease” and 0 for “good SRH for CKD disease.”

### 3.2 Explanatory variables

Socio-demographic characteristics:

This study included demographic information of the respondents such as age groups (18-44 years, 45-59 years, 60-74 years, 75 years and above), gender (male, female), religion (Hindu, Muslim, Christian, others), caste (scheduled caste, scheduled tribe, other backward classes (OBC), others), residence (rural, urban). Additionally, it included social characteristics like education (no education/below primary, primary, secondary, Intermediate, and above), monthly per capita expenditure (MPCE quintile grouped as poorest, poor, middle, richer, and richest), and marital status (having a partner, not having a partner)

Chronic conditions/diseases:

Based on the extensive literature review we included 10 diseases. These are (a) Hypertension/High blood pressure, (b) Diabetes or High blood sugar, (c) Cancer or Malignant tumor, (d) Chronic lung disease (asthma, chronic obstructive pulmonary disease (COPD)/Chronic bronchitis or other chronic lung problems) (e) Chronic heart diseases (Coronary heart disease such as heart attack or Myocardial Infarction), Congestive heart failure, or Other chronic heart problems (f) Stroke (g) Arthritis or rheumatism, Osteoporosis or other bone/joint-related diseases (h) Any neurological, or psychiatric problems such as depression, Alzheimer’s/Dementia, unipolar/bipolar disorders, convulsions, Parkinson’s,etc (i) High cholesterol (j) Other chronic conditions (Thyroid disorder, Gastrointestinal problems (GERD, Constipation, Indigestion, Piles, Peptic Ulcer), (k) Skin diseases. Considering all these above mentioned diseases, a composite indicator for the chronic conditions was developed. It was coded as 0,1,2, and, 3 for “no chronic condition”, “one chronic condition”, “two chronic conditions”, and 3 “three and above chronic conditions” respectively (25, 26).

Health care utilization:

Individuals were asked about their past 12-month health care utilization status in (a) public facility (health post/sub-centers, primary health center/urban health centre, community health center, district/Sub-district hospital, government/tertiary hospital, govt. AYUSH hospital). (b) private facility (private hospital/nursing home, private clinic (OPD-based services), NGO/Charity/Trust/Church-run hospital, private AYUSH hospital) (c) Others (health camp, mobile healthcare unit, pharmacy/drugstore, home visit, Other). Further, it was categorized as visiting hospital facilities, not visting hospital facilities. Those who were availing hospital were again classified based on different health facilities such as public, private, other, any two (public and private) facility visits, and all three (public, private and others) visits, respectively (25).

### 3.3 Statistical analysis

The sociodemographic characteristics of the population with CKD were determined using univariate analysis presented as frequency and proportion. A bivariate analysis employing a chi-square test was carried out to assess the association between self-rated health and CKD with the selected chronic conditions. Lastly, multivariable analysis was utilized with bonferroni correction (given in **Supplementary 1**) to assess the association of socio-demographic characteristics that explains and predicts poor SRH. The marginal probability plot of the interaction between age and chronic condition order on having poor self-rated health is done. The trend of healthcare use among CKD patients was depicted graphically. STATA v.17 software was used for the entire statistical analysis, considering a P ≤ 0.05, and P≤ 0.01 statistically significant. Survey weight was utilized to compensate complex survey design.

### 3.4 Ethical consideration

The data were obtained from IIPS, Mumbai, *via* legitimate methods, and with sufficient authorization. There is no risk to participants because the current study is based on secondary, anonymized data obtained from LASI, the wave-1. The dataset used is duly acknowledged and cited wherever needed.

## 4 Results

The majority (41.18%) of the participants belonged to the 60-74 years age group. Around 17.65% of total participants were on dialysis. About half (51.23%) of the participants were males. Most of the respondents belonged to rural settings (56.55%). Around one-fourth (25.36%) of CKD participants had no formal education. More than three fourth had at least one chronic condition (77.79%). More than half the proportion (63.95%) had rated their health as poor (**Table 1**).

**Table 1:** Background characteristics of chronic kidney disease patients.

Background characteristics	n (%)
Age group	
18-44 years	39 (7.40)
45-59 years	203 (38.52)
60-74 years	217 (41.18)
75 years or above	68 (12.9)
Gender	
Male	270 (51.23)
Female	257 (48.77)
Religion	
Hindu	352 (66.79)
Muslim	55 (10.44)
Christian	106 (20.11)
Other	13 (2.47)
None	1 (0.19)
Education	
No education/below primary	89 (25.36)
Primary completed	78 (22.22)
Secondary/matriculation completed	114 (32.48)
Intermediate and above	70 (19.94)
Caste	
Scheduled caste	81 (16.01)
Scheduled Tribe	122 (24.11)
Other Backward Classes	178 (35.18)
Other	125 (24.70)
Residence	
Rural	298 (56.55)
Urban	229 (43.45)
MPCE quintile*	
Poorest	93 (17.65)
Poorer	76 (14.42)
Middle	103 (19.54)
Richer	122 (23.15)
Richest	133 (25.24)
Working status	
Working	191 (36.24)
Not working	336 (63.76)
Marital Status	
Having a partner	426 (80.83)
Not having partner	101 (19.17)
Chronic conditions	
0	117 (22.20)
1 chronic conditions	153 (29.03)
2 chronic conditions	109 (20.68)
3 and above chronic conditions	148 (28.08)

*MPCE, Mean per capita expenditure quintile.

Identified residence, caste, chronic conditions, and health care utilization were significantly associated with poor SRH. We found aged 75 years and older had a higher likelihood of poor SRH (AOR=1.8, 95% CI= 0.5-6.8). The risk of poor SRH was reported to be higher (AOR 1.8, 95% CI =1.0 -3.1) among the rural population than in the urban population. We observed a higher likelihood of having poor SRH among patients with three and above chronic conditions (AOR=5.1, 95% CI= 2.3-11.0). As the order of chronic conditions increases among CKD patients, there is a sharp inclination to higher chances of poor SRH (**Table 2**).

**Table 2:** Multivariable logistic regression model of self-rated health and CKD patients with their background characteristics.

Background characteristics	CKD with Good SRH n=186 n (%)	CKD with Poor SRH n=330 n (%)	Adjustedodds ratio (95% CI)
Age			
18-44 years	17 (9.14)	22 (6.67)	1
45-59 years	84 (45.16)	117 (35.45)	0.9 (0.4 - 2.3)
60-74 years	67 (36.02)	146 (44.24)	1.4 (0.5 - 3.7)
75 years & above	18 (9.68)	45 (13.64)	1.8 (0.5 - 6.8)
Gender			
Male	94 (50.54)	170 (51.52)	1
Female	92 (49.46)	160 (48.48)	1.1 (0.5 - 2.1)
Residence			
Urban	95 (51.08)	196 (59.39)	1
Rural	91 (48.92)	134 (40.61)	1.8 (1.0 - 3.1)**
Religion			
Hindu	123 (66.13)	221 (66.97)	1
Muslim	13 (6.99)	40 (12.12)	0.7 (0.2 - 2.0)
Christian	44 (23.66)	61 (18.48)	1.5 (0.6 - 3.9)
Other	5 (2.69)	8 (2.42)	1.4 (0.3 - 6.0)
None	1 (0.54)	0	
Caste			
Scheduled caste	35 (19.55)	44 (13.88)	1
Scheduled tribe	52 (29.05)	69 (21.77)	2.3 (0.8 - 6.6)
Other Backward Class	51 (28.49)	121 (38.17)	2.4 (1.0 - 5.5)**
Other	41 (22.91)	83 (26.18)	1.9 (0.8 - 4.5)
Education			
No education/below primary	28 (21.54)	60 (27.52)	1
Primary completed	32 (24.62)	46 (21.10)	0.8 (0.4 - 1.7)
Secondary/matriculation completed	47 (36.15)	66 (30.28)	0.8 (0.4 - 1.5)
Intermediate and above	23 (17.69)	46 (21.10)	0.9 (0.4 - 2.3)
Working status			
Working	87 (46.77)	102 (30.91)	1
Not-working	99 (53.23)	228 (69.09)	1.1 (0.6 - 1.9)
MPCE			
Rich	84 (45.16)	168 (50.91)	1
Middle	40 (21.51)	61 (18.48)	0.7 (0.4 - 1.4)
Poor	62 (33.33)	101 (30.61)	1.1 (0.6 - 2.0)
Marital status			
Not having partner	36 (19.35)	62 (18.79)	1
Having partner	150 (80.65)	268 (81.21)	1.5 (0.7 - 3.0)
Chronic conditions			
0	63 (33.87)	50 (15.15)	1
1	63 (33.87)	87 (26.36)	2.1 (1.1 - 4.2)**
2	29 (15.59)	79 (23.94)	3.5 (1.6 - 7.5)***
3 and above	31 (16.67)	114 (34.55)	5.1 (2.3 - 11.0)***
Health care utilization			
Not availing hospital	58 (31.18)	49 (14.94)	1
Availing hospital	128 (68.82)	279 (85.06)	2.3 (1.2 - 4.6)***

**P ≤ 0.05, ***P ≤ 0.01, CI, Confidence interval.

**Figure 1:**
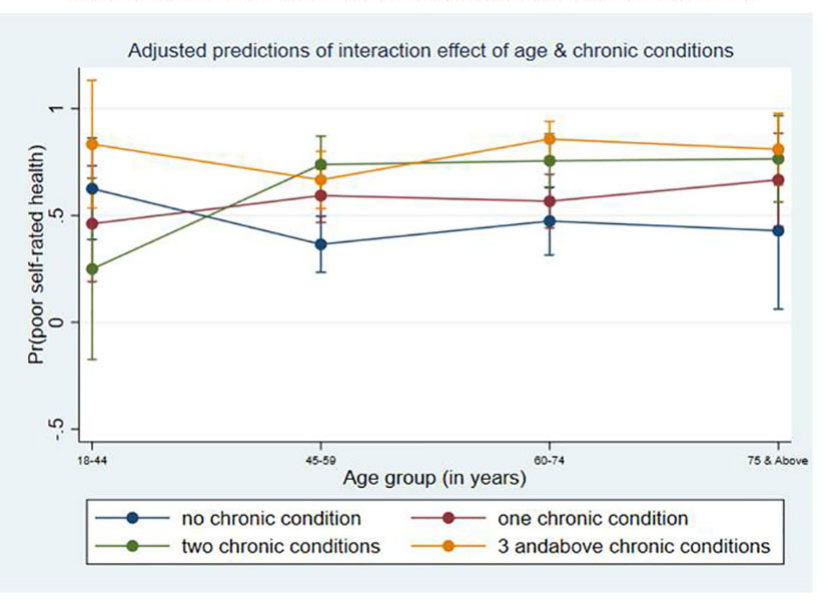
Adjusted prediction interaction effect of age and chronic conditions.

The marginal probability plot of the interaction between age and chronic condition order on having poor self-rated health is shown in **Figure 1**. CKD patients aged 75 years and older with no chronic condition had a probability of having a poor SRH of 0.43. CKD patients aged 75 years and older with one chronic condition had a probability of having poor health of 0.67; two chronic conditions had a likelihood of having poor health of 0.76, and three or more chronic conditions had a probability of being in poor health of 0.81. Consequently, it was observed that the likelihood of having poor SRH increases with an increase in the order of the chronic conditions as the age increases. (Estimated value described **Supplementary 2**)

**Figure 2:**
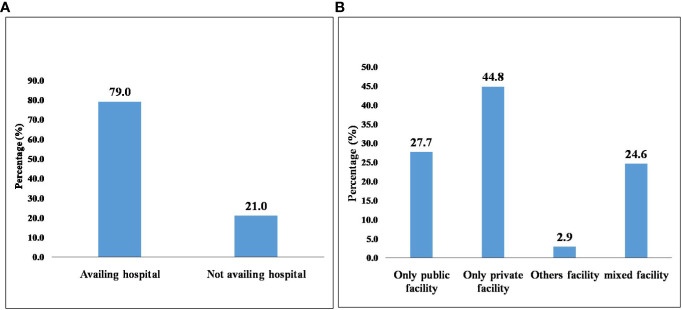
Health care utilization (HCU) among CKD patients in India. **(A)** Health care utilization among CKD patients. **(B)** Various health care facilities accessed by CKD patients.

Among all CKD patients, 415 (79%) individuals had visited health care, while 110 (21%) individuals did not visit health care. Most of the participants who availed of health care sought care from a private hospital (44.8%), while 27.7% of the participants sought care from public or government hospitals (**Figure 2**).

The reasons for not accessing health care among individuals were associated with various reasons. Of those individuals who did not avail services from any facility, 62% of the participants reported not falling sick or having any symptoms of CKD. A detailed description was provided in **Supplementary 3**.

We observed a significantly higher proportion reported their health to be fair (**Table 3**).

**Table 3:** Self-rated health profile of CKD with chronic conditions/ disease status among (18+) adult population in India (LASI) wave 1, 2017-18.

Self-rated health	CRF with chronic conditions/diseases status		χ2 Value (P-Value)
	Present (n=410) n(%)	Absent (n= 117) n(%)	
Excellent	8 (1.99)	4 (3.54)	35.74 (<0.01)
Very good	29 (7.20)	15 (13.27)	
Good	86 (21.34)	44 (38.94)	
Fair	153 (37.97)	42 (37.17)	
Poor	127 (31.51)	8 (7.08)	

## 5 Discussion

The current study used nationally representative data to assess the factors associated with poor SRH among CKD patients and their health care utilization. Participants aged 75 years and above, those who lived in rural areas, and who had one or more chronic diseases were reported to increase the likelihood of poor SRH. The interaction between ageing and the increased number of chronic diseases led to poor SRH. The majority of CKD patients visited hospitals. Compared to public hospitals, the majority of those visits went to private hospitals.

Participants aged 75 years and above had a higher likelihood of having poor SRH which is consistent with the findings of a study conducted among CKD patients in India. This study found older adults (50 years and older) are associated with physical and mental component summary which are the key indicators of quality of life (27). A probable explanation for this could be the older adults rely on others for financial support while seeking medical treatment (28). Additionally, due to their low income and status as a member of a socially deprived group, older people may be given lower priority (28). Vulnerability of elder adults due to their financial and physical dependency is well known. Our findings also stated that rural residents were also associated with poor SRH. It could be linked to the unavailability of medical resources. For example, it was found only 33% of all health professionals work in rural India, although this region’s population accounted for roughly 66% of the country’s total in 2018 (13). Also, data stated doctor and nurse populations are only 27% and 36%, respectively, in rural areas (13). Additionally, age, chronic conditions, and SRH are three interlinked factors and also interact with each other. Our findings also revealed that poor SRH would be the interactive effect of increased age and increase in the number of chronic conditions.

The current study depicted health visit was found to be high among CKD patients. Most of CKD individuals availed private facilities (44.8%) than public (27.7%) and mixed facilities (24%), which may have attributed to long waiting hours, long queues, poor quality of care, unavailability of doctors, insufficient time to seek care, and cost, significantly in a public setting (29, 30). All the findings could also be relate to the fact that the private sector employs most of the entire health staff and provides about 60% of inpatient and 70% of outpatient care (13). However, it was revealed those seeking private medical treatment had a 16 times higher likelihood of incurring catastrophic health expenditures (CHE) than those seeking public facilities (14), which could be result to improvishment in the care seeker. Hence those are affluent or economically sound could only avail private facilities (13, 14). Additionally, individual attributable factors like caste, education, and wealth quintile are the main factors determining the choice of selecting the private sector rather than the government (29), which could be a reason for selecting a private setting.

Again, it found that patients (2.9%) seek care from other facilities like health camps, mobile health care units, dispensaries, home visits, anganawadi centers, etc., which could be a window of opportunity to aware them about the importance of health visits. This study indicated that around 21% of CKD patients were not receiving health care. The majority stated the main reason for not availing of HCU was many. These were not getting sick, having to get involved in the occupational work or dependency on family members for availing services, taking treatment at home, or having the involvement of family members whose decision was necessary for availing services.

### 5.1 Implication of policy and practice

Therefore, it is necessary to facilitate general services such as early awareness and screening programs. Strengthening health care services, framing policies and programs for health coverage among the CKD population, and empowering the head of the family educationally and financially would help combat such issues in the country. It also necessitate equitable and strengthen the existing health system.

### 5.2 Strength and limitation of the study

The current study’s findings were based on the nationwide survey, LASI. This is one of the few study highlighting to the factor associated with SRH among CKD patients. Moreover, it utilized the socioeconomic parameters and the NCDs data to create statistical evidence for the pattern of health care utilization among CKD patients. The study, however, is based on self-reported data, which may undermine the true prevalence. To project causal inference, the temporality of the factors was not known as it is cross-sectional survey.

## 6 Conclusion

We observed a higher prevalence of poor self-rated health among CKD patients. Older adults, rural residents and presence of other chronic conditions were identified to be associated with poor self-rated health. Evidences necessitate strengthening and proper decentralizing of health system. It also indicates the urgent need for equitable, affordable and quality health care services.

## Data availability statement

Publicly available datasets were analyzed in this study. This data can be found here: https://www.iipsindia.ac.in/content/lasi-wave-i.

## Ethics statement

The studies involving human participants were reviewed and approved by Indian Council of Medical Research (ICMR) and IIPS, Mumbai. The patients/participants provided their written informed consent to participate in this study.

## Author contributions

The study was planned by the corresponding author SP and SK, who also served as the study’s overall supervisor. SRN, SN, and SK were active in data analysis and variable identification. SN, SRN, and AA were all active in the paper writing process. SN was in charge of paper drafting and editing. All authors contributed to the article and approved the submitted version.
