# Supplementary material for: Factors associated with poor self-rated health among chronic kidney disease patients and their health care utilization: Insights from LASI wave-1, 2017-18
Source: Front Nephrol. 2023 Jan 6;2:968285. doi: 10.3389/fneph.2022.968285 (PMC10479761; doi:10.3389/fneph.2022.968285)
Supplement: Supplementary file 1 [file Table_1.docx]

**Supplementary-1: Bonferroni estimate of explanatory variables with p-values.**

| **Variables** | **Contrast** | **SE** | **z- value** | **P- value** | **95% Confidence interval** | |
| --- | --- | --- | --- | --- | --- | --- |
| **Age** |  |  |  |  |  |  |
| 45-59 vs 18-44 | -0.06 | 0.46 | -0.13 | 1.00 | -1.27 | 1.15 |
| 60-74 vs 18-44 | 0.32 | 0.52 | 0.61 | 1.00 | -1.04 | 1.68 |
| 75 & Above vs 18-44 | 0.57 | 0.69 | 0.83 | 1.00 | -1.24 | 2.39 |
| 60-74 vs 45-59 | 0.38 | 0.33 | 1.16 | 1.00 | -0.48 | 1.24 |
| 75 & Above vs 45-59 | 0.63 | 0.55 | 1.16 | 1.00 | -0.80 | 2.07 |
| 75 & Above vs 60-74 | 0.26 | 0.49 | 0.53 | 1.00 | -1.02 | 1.54 |
| **Gender** |  |  |  |  |  |  |
| Female vs male | 0.068 | 0.35 | 0.19 | 0.85 | -0.62 | 0.76 |
| **Residence** |  |  |  |  |  |  |
| Urban vs Rural | -0.58 | 0.28 | -2.10 | 0.04 | -1.13 | -0.04 |
| **Religion** |  |  |  |  |  |  |
| Muslim vs Hindu | -0.34 | 0.54 | -0.64 | 1.00 | -1.85 | 1.16 |
| Christian vs Hindu | 0.39 | 0.49 | 0.79 | 1.00 | -1.00 | 1.78 |
| Other vs Hindu | 0.35 | 0.74 | 0.47 | 1.00 | -1.72 | 2.42 |
| None vs Hindu | Not estimated | | | | | |
| Christian vs Muslim | 0.73 | 0.68 | 1.07 | 1.00 | -1.18 | 2.65 |
| Other vs Muslim | 0.69 | 0.89 | 0.77 | 1.00 | -1.82 | 3.20 |
| None vs Muslim |  |  |  |  |  |  |
| Other vs Christian | -0.05 | 0.85 | -0.05 | 1.00 | -2.44 | 2.35 |
| None vs Christian | Not estimated | | | | | |
| None vs Other | Not estimated | | | | | |
| **Caste** |  |  |  |  |  |  |
| Scheduled tribe vs Scheduled caste | 0.83 | 0.54 | 1.52 | 0.77 | -0.61 | 2.26 |
| OBC vs Scheduled caste | 0.88 | 0.43 | 2.06 | 0.24 | -0.25 | 2.00 |
| Other vs Scheduled caste | 0.65 | 0.43 | 1.49 | 0.82 | -0.50 | 1.79 |
| OBC vs Scheduled tribe | 0.05 | 0.49 | 0.10 | 1.00 | -1.24 | 1.34 |
| Other vs Scheduled tribe | -0.18 | 0.51 | -0.36 | 1.00 | -1.53 | 1.17 |
| Other vs OBC | -0.23 | 0.36 | -0.64 | 1.00 | -1.19 | 0.72 |
| **Education** |  |  |  |  |  |  |
| Primary completed vs No education/primary not completed | -0.19 | 0.38 | -0.50 | 1.00 | -1.19 | 0.81 |
| Secondary completed vs No education/primary not completed | -0.28 | 0.37 | -0.76 | 1.00 | -1.24 | 0.68 |
| Higher and above vs No education/primary not completed | -0.02 | 0.43 | -0.05 | 1.00 | -1.16 | 1.12 |
| Secondary completed vs Primary completed | -0.09 | 0.36 | -0.25 | 1.00 | -1.03 | 0.85 |
| Higher and above vs Primary completed | 0.17 | 0.41 | 0.41 | 1.00 | -0.92 | 1.26 |
| Higher and above vs Secondary completed | 0.26 | 0.38 | 0.67 | 1.00 | -0.75 | 1.26 |
| **Working status** |  |  |  |  |  |  |
| not working vs working | 0.05 | 0.30 | 0.15 | 0.88 | -0.54 | 0.63 |
| **wealth quintile** |  |  |  |  |  |  |
| Middle vs Poor | -0.42 | 0.37 | -1.13 | 0.77 | -1.30 | 0.46 |
| Rich vs Poor | -0.07 | 0.31 | -0.22 | 1.00 | -0.82 | 0.68 |
| Rich vs Middle | 0.35 | 0.33 | 1.05 | 0.88 | -0.45 | 1.15 |
| **Marital status** |  |  |  |  |  |  |
| Not having a partner vs having a partner | -0.40 | 0.36 | -1.11 | 0.27 | -1.11 | 0.31 |
| **Morbidity** |  |  |  |  |  |  |
| one morbidity vs no morbidity | 0.78 | 0.36 | 2.18 | 0.18 | -0.16 | 1.71 |
| two morbidity vs no morbidity | 1.27 | 0.39 | 3.23 | 0.01 | 0.23 | 2.30 |
| 3 and above morbidity vs no morbidity | 1.62 | 0.40 | 4.09 | 0.00 | 0.58 | 2.67 |
| two morbidity vs one morbidity | 0.49 | 0.37 | 1.32 | 1.00 | -0.49 | 1.48 |
| 3 and above morbidity vs one morbidity | 0.85 | 0.37 | 2.30 | 0.13 | -0.13 | 1.82 |
| 3 and above morbidity vs two morbidity | 0.35 | 0.38 | 0.93 | 1.00 | -0.66 | 1.36 |
| **Any health care** |  |  |  |  |  |  |
| Not having a health care vs availing health care | -0.85 | 0.34 | -2.49 | 0.01 | -1.52 | -0.18 |
