# Supplementary material for: Factors associated with poor self-rated health among chronic kidney disease patients and their health care utilization: Insights from LASI wave-1, 2017-18
Source: Front Nephrol. 2023 Jan 6;2:968285. doi: 10.3389/fneph.2022.968285 (PMC10479761; doi:10.3389/fneph.2022.968285)
Supplement: Supplementary file 2 [file Table_2.docx]

**Supplementary-2:**

**(A) Binary logistic regression of Poor self-rated health with interaction of age-comorbidity.**

| **Interaction variables** | **Odds Ratio** | **P-value** | **95% Confidence interval** | |
| --- | --- | --- | --- | --- |
| Age x co-morbidity |  |  |  |  |
| 18-44 x no morbidity | Ref. |  |  |  |
| 18-44 x one morbidity | 0.51 | 0.38 | 0.12 | 2.28 |
| 18-44 x two morbidity | 0.20 | 0.20 | 0.02 | 2.39 |
| 18-44 x 3 and above morbidity | 3.00 | 0.36 | 0.28 | 32.21 |
| 45-59 x no morbidity | 0.35 | 0.07 | 0.11 | 1.10 |
| 45-59 x one morbidity | 0.88 | 0.82 | 0.28 | 2.73 |
| 45-59 x two morbidity | 1.69 | 0.40 | 0.50 | 5.75 |
| 45-59 x 3 and above morbidity | 1.20 | 0.76 | 0.37 | 3.89 |
| 60-74 x no morbidity | 0.54 | 0.31 | 0.16 | 1.79 |
| 60-74 x one morbidity | 0.78 | 0.68 | 0.25 | 2.44 |
| 60-74 x two morbidity | 1.85 | 0.32 | 0.55 | 6.28 |
| 60-74 x 3 and above morbidity | 3.60 | 0.04 | 1.07 | 12.11 |
| 75 & Above x no morbidity | 0.45 | 0.39 | 0.07 | 2.74 |
| 75 & Above x one morbidity | 1.20 | 0.80 | 0.29 | 4.91 |
| 75 & Above x two morbidity | 1.95 | 0.39 | 0.43 | 8.83 |
| 75 & Above x 3 and above morbidity | 2.55 | 0.22 | 0.58 | 11.28 |

**(B) Marginal effect of poor self-rated health with interaction of age-comorbidity.**

| **Interaction variables** | **Delta-method** | | | |
| --- | --- | --- | --- | --- |
| **Age x co-morbidity** | **Marginal** | **P- value** | **95% Confidence interval** | |
| 18-44 x no morbidity | 0.63 | 0.0 | 0.39 | 0.86 |
| 18-44 x one morbidity | 0.46 | 0.0 | 0.19 | 0.73 |
| 18-44 x two morbidity | 0.25 | 0.2 | -0.17 | 0.67 |
| 18-44 x 3 and above morbidity | 0.83 | 0.0 | 0.54 | 1.13 |
| 45-59 x no morbidity | 0.37 | 0.0 | 0.23 | 0.50 |
| 45-59 x one morbidity | 0.59 | 0.0 | 0.47 | 0.72 |
| 45-59 x two morbidity | 0.74 | 0.0 | 0.61 | 0.87 |
| 45-59 x 3 and above morbidity | 0.67 | 0.0 | 0.53 | 0.80 |
| 60-74 x no morbidity | 0.47 | 0.0 | 0.31 | 0.63 |
| 60-74 x one morbidity | 0.57 | 0.0 | 0.44 | 0.69 |
| 60-74 x two morbidity | 0.76 | 0.0 | 0.63 | 0.88 |
| 60-74 x 3 and above morbidity | 0.86 | 0.0 | 0.78 | 0.94 |
| 75 & Above x no morbidity | 0.43 | 0.0 | 0.06 | 0.80 |
| 75 & Above x one morbidity | 0.67 | 0.0 | 0.45 | 0.88 |
| 75 & Above x two morbidity | 0.76 | 0.0 | 0.56 | 0.97 |
| 75 & Above x 3 and above morbidity | 0.81 | 0.0 | 0.64 | 0.98 |
