# Supplementary material for: Factors associated with poor self-rated health among chronic kidney disease patients and their health care utilization: Insights from LASI wave-1, 2017-18
Source: Front Nephrol. 2023 Jan 6;2:968285. doi: 10.3389/fneph.2022.968285 (PMC10479761; doi:10.3389/fneph.2022.968285)
Supplement: Supplementary file 3 [file Table_3.docx]

**Supplementary 3: Main reasons for not accessing health care utilization**

| **Sl no:** | **Main reasons** | **n=97 n (%)** |
| --- | --- | --- |
| 1. | Did not get sick | 60 (61.86) |
| 2. | Needed to work | 2 (2.06) |
| 3. | Did not want to give up a day's work | 1 (1.03) |
| 4. | Did not have enough money/cost was too high | 9 (9.28) |
| 5. | Treatment was unlikely to be effective | 2 (2.06) |
| 6. | Illness was not considered serious | 14 (14.43) |
| 7. | Had no one to accompany | 2 (2.06) |
| 8. | Had medicine at home | 6 (6.19) |
| 9. | Family member(s) decided it was not required | 1 (1.03) |
